# Is it best on the nest? Effects of avian life-history on haemosporidian parasitism

**DOI:** 10.1016/j.ijppaw.2020.07.014

**Published:** 2020-07-31

**Authors:** Claudia Ganser, Ara Monadjem, Robert A. McCleery, Thandeka Ndlela, Samantha M. Wisely

**Affiliations:** a Department of Wildlife Ecology and Conservation, 110 Newins-Ziegler Hall, University of Florida, Gainesville, FL, 32611, USA; b Department of Biological Sciences, University of Eswatini, Kwaluseni, Eswatini; c Mammal Research Institute, Department of Zoology & Entomology, University of Pretoria, Pretoria, South Africa

**Keywords:** Avian malaria, *Plasmodium*, Life-history, Haemosporida, Africa, Nest

## Abstract

Infectious diseases vary in prevalence and pathology among host species. Species may differ in prevalence of infection due to varying exposure and susceptibility to disease agents throughout their lifetime, which may be attributable to underlying differences in their phenology, physiology and behavior. A recently growing body of literature has focused on the utility of host life-history traits to provide mechanistic explanations for interspecific variation in host-parasite associations. In this study, we utilized diverse avian and haemosporidian assemblages in an African savanna to evaluate the link between haemosporidia prevalence (*Plasmodium*, *Haemoproteus*, *Leucocytozoon*) and avian life-history traits such as body size, mating system, nest care and nest structure. We found that variation of haemosporidia prevalence was consistent with life-history traits that pertain to the reproduction of avian host. Nest care was the single most important predictor of infection status. In birds with shared and female-only nest care, the expected rates of parasitism were between 8- and 12-fold higher than in avian brood parasites that provide no nest care. This finding supports the hypothesis that parental care is an evolutionarily costly life-history trait that increases species' risk of infection with vector-borne diseases. The influence of other host traits (nest structure, body size) was less consistent suggesting that differences in the vectors’ ecology and host-seeking behavior produce variable patterns of parasitism among haemosporidia genera. Nest structure influenced infection with *Haemoproteus* and *Leucocytozoon* only. *Leucocytozoon* infections were associated with ground-nesting birds, while *Haemoproteus* infections were associated with birds that build open nest structures. Body size was an important predictor of *Leucocytozoon* infections, particularly large-bodied birds like guineafowl and doves, which exhibited high prevalences.

## Highlights

• Variation in infection prevalence was consistent with reproductive traits in avian hosts.
• Avian species that invested less in nest care had lower infection rates than species with other nest care strategies.
• *Leucocytozoon* infections were high in ground-nesting birds; birds with open nests had higher *Haemoproteus* infections.
• Birds like guineafowl and doves with larger body size had more *Leucocytozoon* infections.

## Graphical abstract

**Figure:**
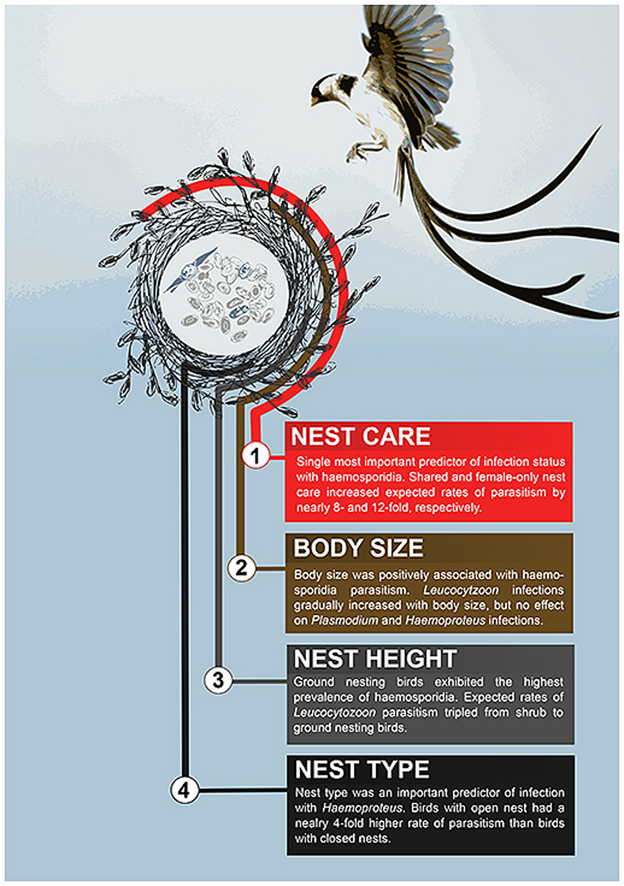

## 1 Introduction

Identifying factors that contribute to variation in disease risk promotes understanding of the ecological and evolutionary processes that drive host-parasite associations and may have important implications for human health (Atkinson and van Riper, 1991). Traits of importance, particularly for zoonotic pathogens, are those that affect prevalence and virulence within populations as well as across taxa. Avian models have helped to shape our understanding of host factors that mediate the epidemiology of pathogens (Ostfeld and Kessing, 2000; Kilpatrick et al., 2006; Cronin et al., 2010). Exploration into the role of life-history traits have allowed us to identify factors that facilitate the transmission of arboviruses (West Nile virus: Kilpatrick et al., 2006; Janousek et al., 2014) and bacteria (*Borrelia burgdorferi*: Giardina et al., 2000; Ogden et al., 2008).

A growing body of literature has shown how host life-history traits explain interspecific variation in host-parasite associations and provide a mechanistic understanding for observed differences in rates of parasitism. Indeed, evidence from across the animal kingdom (amphibians: Todd, 2007; birds: Arriero and Moller, 2008; mammals: Jones et al., 2008) suggests linkages between host life-history traits and patterns of pathogen prevalence. In avian systems, the prevalence and susceptibility of pathogens may vary significantly between host species even for those that co-occur sympatrically or even syntopically. For instance, following the introduction of avian malaria to the Hawaiian Islands, the prevalence of malaria in native avifauna varied greatly between species from 2.1% in Omao (*Myadestes obscurus*) to 29.2% in Apapane (*Himatione sanguinea*, van Riper et al., 1986). Species may differ in prevalence of infection due to varying exposure and susceptibility to disease agents throughout their lifetime. Some of this variation may be attributable to underlying differences in their phenology, physiology and behavior (Lee et al., 2008; Cronin et al., 2010). For instance, transmission of avian influenza viruses (AIV) in European waterfowl is affected by the sociality of species (e.g., degree of aggregation; Munster et al., 2006), and avian roosting behavior has been shown to affect West Nile virus (WNV) infections in passerine hosts (Janousek et al., 2014).

Avian malaria and other related haemosporidia are a common and widespread group of vector-borne parasites comprising three genetically diverse genera: *Plasmodium*, *Haemoproteus*, and *Leucocytozoon*. Avian haemosporidia provide insight into life-history correlates of host-parasite interactions because they often infect multiple host species, and prevalence as well as disease severity varies substantially among them (Ewald, 1983; Poulin, 2007). These parasites have served as model systems of host-parasite associations (Ricklefs, 1992; Garvin and Remsen, 1997) and parasite-mediated selection processes (Hamilton and Zuk, 1982; Read, 1988; Scheuerlein and Ricklefs, 2004), and have provided insight into the epidemiology of human malaria (Jasinskiene et al., 2007).

Two observations suggest that haemosporidia have influenced the evolution of avian life history traits. The first observation involves the immune system, reproduction, and infection status. During the acute phase of haemosporidia infection, host fitness and survival may be compromised (Ferrell et al., 2007; Lachish et al., 2011), and surviving individuals then enter the chronic phase of infection. Because chronic infections persist with low parasitemia, they can contribute to the further transmission of these parasites (Valkiunas, 2005). During reproduction, birds become immunosuppressed allowing chronic infections to relapse to acute infections (Valkiunas, 2005). Because parental effort can downregulate immune function (Faivre et al., 2003, Alonso-Alvarez et al., 2004), increased reproductive effort may drive the evolution of low investment in reproduction. Hence, animals that invest less in reproduction such as brood parasites may be able to maintain sufficient immune function and suffer fewer bouts of acute infection and thereby increasing their lifetime fitness. The second way in which haemosporidia have influenced avian life-history traits is via exposure and susceptibility to bites from arthropod vectors (Valkiunas, 2005; Table S1). Timing of breeding and nesting behavior influences vulnerability of nesting birds to parasitism (Blackmore and Dow, 1958; Burkett-Cadena et al., 2010). Breeding that coincides with a high abundance of vectors and elevated intrinsic replication of parasites with vector species may increase their distribution and exposure rates to avian hosts. For instance, Fecchio et al. (2017) found that the distribution of *Plasmodium* parasites might be constrained by the abundance of mosquitoes. Differences in allocation to nest care may affect parasitism as well, such that individuals that provide limited care experience lower exposure and susceptibility to infection (Richner et al., 1995). For example, individuals or species that allocate more resources to caring for their offspring become susceptible due to compromised immunity as a trade-off or increased exposure due to prolonged periods on the nest.

The structure of the nest itself (specifically nest height or nest type) may provide differential protection to host-seeking dipterans depending on the niche partitioning of the vector species (Fecchio et al., 2011; Svensson-Coelho et al., 2013; Lutz et al., 2015; Matthews et al., 2016). Because haemosporidia are vectored by multiple genera of dipterans with varying habitat requirements (*Plasmodium*: Černŷ et al., 2011; *Haemoproteus*: Garvin and Geiner, 2003, McGregor et al., 2018; *Leucocytozoon*: Rohner et al., 2000), nest height and structure may be important mediators of host exposure. For instance, in south-central Africa, closed cup nesting birds tended to have a higher prevalence of mosquito-transmitted *Plasmodium* parasites than birds with open nests (Lutz et al., 2015). In contrast, *Haemoproteus* parasites that were commonly transmitted by biting midges exhibited a higher prevalence in birds with open than closed nests (Lutz et al., 2015). Although nest characteristics have been used to address heterogeneity in prevalence among host species, results have been mixed. Review of the literature suggests that use of nests as an indicator of exposure risk may largely be dependent on host-vector contact rates and environment (Fecchio et al., 2017). Host body size may further affect rates of vector parasitism since larger-bodied birds release more olfactory cues (Scheuerlein and Ricklefs, 2004) and have greater surface area for biting dipterans (Hamilton and Zuk, 1982). Overall rates of parasitism and sex differences in infection probability may be further mediated by mating systems (Zuk, 1996; Hillgarth et al., 1997). For example, pair-bonded species (monogamous) exhibit less sex-based differences in infection probability than species where males mate with multiple females (polygynous) (Richner et al., 1995).

In this study, we investigated the association between life-history traits and prevalence of three haemosporidian genera in savanna birds of Eswatini. Differences in host morphology, variability in behavior and endocrinological responses associated with reproduction, in particular nesting, affect host-vector encounter rates that can result in differential rates of haemosporidian parasitism within avian communities (Lutz et al., 2015). Specifically, we aimed to: (1) describe the prevalence patterns of the three most common haemosporidia genera (*Plasmodium*, *Haemoproteus*, *Leucocytozoon*) to uncover evidence of complex host-parasite interactions; and (2) test the hypothesis that life-history traits such as body size, mating system, nest care and nest structure (nest type and nest height) not only give rise to observed patterns of host-parasite interactions; but also (3) predict parasitism rates by the different haemosporidia genera each of which is transmitted by a different vector species.

## 2 Materials and methods

### 2.1 Field sites

**Fig. 1:**
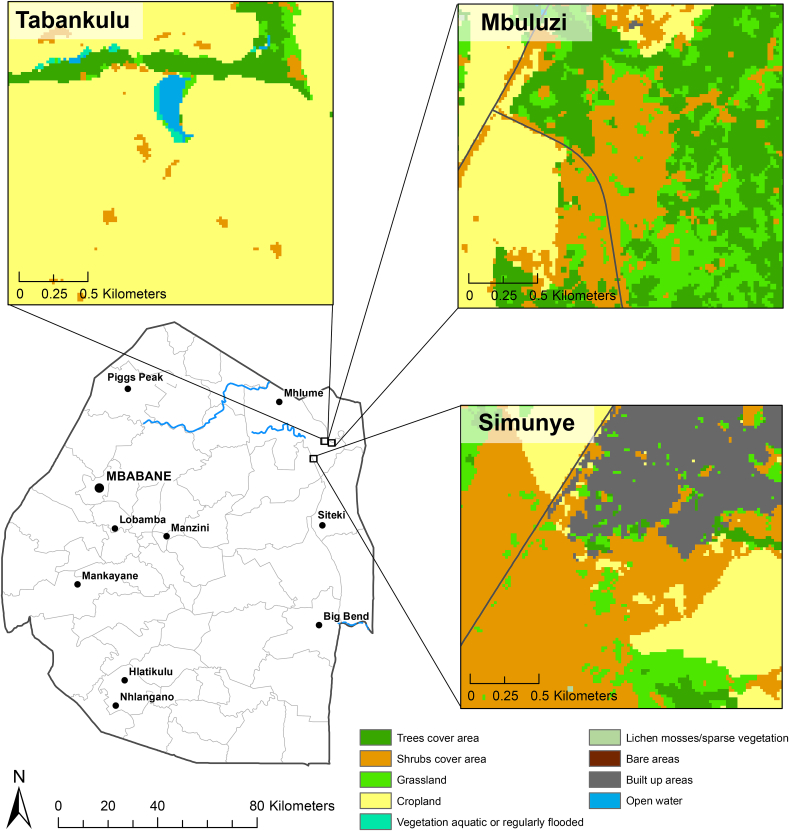
Locations and land cover of sampling sites of Tabankulu village, Simunye town, and Mbuluzi Game Reserve in northeastern Eswatini (Eswatini Sentinel2 Land Use Land Cover 2016).

Field sites were located in northeastern Eswatini across different land-use types: Mbuluzi Game Reserve (26°09′21.6″S, 31°59′04.1″E), Simunye town (26°12′50.4″S, 31°55′10.9″E), and Tabankulu village (26°08′58.6″S, 31°57′36.5″E, Fig. 1). Mbuluzi Game Reserve is a conservation area that is explicitly managed for the conservation of wildlife (Hurst et al., 2013). Simunye town borders conservation areas and is characterized by peri-urban land-use, while Tabankulu village is located on a large-scale sugar cane plantation. Overall, the landscape is characterized by a complex mosaic of sugarcane plantations, conservation areas, and lands managed for wildlife conservation and sustainable grazing (Monadjem and Garcelon, 2005). The eastern portion of the country is the warmest and driest region in Eswatini, with a subtropical climate and distinct wet (October–March) and dry (April–September) seasons. Annual precipitation ranges between 550 and 725 mm (Motando et al., 2004, 2005). The moist climate and diverse landscape of northeastern Eswatini likely increases diversity and density of avian species (Monadjem, 2005). Nearly 300 species of birds from various guilds have been reported in this region (Parker, 1994). Common granivorous birds in northeastern Eswatini are spread across multiple families, and include weavers (Ploceidae), indigobirds (Viduidae), guineafowl (Numidae) and doves (Columbidae; Table 1). Their breeding phenology coincides with the wet season, during which environmental conditions necessary for the proliferation of dipteran vectors are satisfied (Keith, 1992; Urban, 1986; Fry, 1992; Tarboton, 2001). For example, Culicine mosquitoes, vectors of *Plasmodium*, require ephemeral breeding sites, such as standing and stagnant pools of water (Fisher and Schweigmann, 2004; Patz and Olson, 2006). The vectors of *Haemoproteus*, Hippoboscid flies (Hippoboscidae) and biting midges (Ceratopogonidae) require moist soils or water to lay eggs, while black flies that transmit *Leucocytozoon* parasites (Simuliidae) depend on flowing water for breeding (Earlé et al., 1992).

**Table 1:** Prevalence of haemosporidia by species of granivorous bird in Eswatini. Table indicates *Plasmodium*, *Haemoproteus* and *Leucocytozoon*, and overall prevalence estimates (Wilson score interval; Brown et al., 2001).

Family	Scientific name	Common name	Nsamp^a^	*Plasmodium*		*Haemoproteus*		*Leucocytozoon*		*Overall*	
				Npos^b^	% Prev (LCI, UCI)^c^	Npos	% Prev (LCI, UCI)	Npos	% Prev (LCI, UCI)	Npos	% Prev (LCI, UCI)
Estrildidae	*Lagonosticta rubricata*	African Firefinch	3	0	0.00 (0.00, 56.15)	0	0.00 (0.00, 56.15)	0	0.00 (0.00, 56.15)	0	0.00 (0.00, 56.15)
Estrildidae	*Uraeginthus angolensis*	Blue Waxbill	34	3	8.82 (3.05, 22.96)	17	50.00 (34.07, 65, 93)	12	35.29 (21.49, 52.09)	30	88.24 (73.38, 95.33)
Estrildidae	*Lonchura cucullata*	Bronze Mannikin	20	1	5.00 (0.89, 23.61)	2	10.00 (2.79, 30.10)	0	0.00 (0.00, 16.11)	3	15.00 (5.24, 36.04)
Estrildidae	*Lagonosticta senegala*	Red-billed Firefinch	5	2	40.00 (11.76, 76.93)	0	0.00 (0.00, 43.45)	0	0.00 (0.00, 43.45)	2	40.00 (11.76, 76.93)
Ploceidae	*Euplectes axillaris*	Fan-tailed Widowbird	30	24	80.00 (62.69, 90.49)	2	6.67 (1.85, 21.32)	6	20.00 (9.51, 37.31)	28	93.33 (78.68, 98.15)
Ploceidae	*Euplectes ardens*	Red-collared Widowbird	2	2	100.00 (34.24, 100.00)	0	0.00 (0.00, 65.76)	0	0.00 (0.00, 65.76)	2	100.00 (34.24, 100.00)
Ploceidae	*Euplectes orix*	Red Bishop	7	5	71.43 (35.89, 91.78)	1	14.29 (2.57, 51.31)	1	14.29 (2.57, 51.31)	6	85.71 (48.69, 97.43)
Ploceidae	*Ploceus cucullatus*	Village Weaver	129	79	61.24 (52.62, 69.21)	36	27.91 (20.89, 36.20)	47	36.43 (28.63, 45.02)	117	90.70 (84.44, 94.60)
Viduidae	*Vidua paradisaea*	Long-tailed Paradise Whydah	1	1	100.00% (20.56, 100.00)	0	0.00 (0.00, 79.35)	0	0.00 (0.00, 79.35)	1	100.00% (20.56, 100.00)
Viduidae	*Vidua macroura*	Pin-tailed Whydah	35	0	0.00 (0.00, 9.89)	1	2.86 (0.51, 14.53)	1	2.86 (0.51, 14.53)	2	5.71 (2.58, 18.61)
Viduidae	*Vidua funerea*	Dusky Indigobird	75	1	1.32 (0.24, 7.17)	2	2.67 (0.73, 9.21)	3	4.00 (1.37, 11.11)	5	6.67 (2.88, 14.68)
Viduidae	*Vidua chalybeata*	Village Indigobird	9	1	11.11 (1.99, 34.50)	0	0.00 (0.00, 29.91)	0	0.00 (0.00, 29.91)	1	11.11 (1.99, 34.50)
Columbidae	*Streptopelia capicola*	Cape Turtle Dove	1	0	0.00 (0.00, 79.35)	1	100.00 (20.65, 100.00)	0	0.00 (0.00, 79.35)	1	100.00 (20.65, 100.00)
Columbidae	*Turtur chalcospilos*	Emerald-spotted Wood Dove	8	0	0.00 (0.00, 32.44)	8	100.00 (67.56, 100.00)	4	50.00 (21.52, 78.48)	8	100.00 (67.56, 100.00)
Columbidae	*Streptopelia semitorquata*	Red-eyed Dove	15	0	0.00 (0.00, 20.39)	15	100.00 (79.61, 100.00)	8	53.33 (30.12, 75.19)	15	100.00 (79.61, 100.00)
Numidae	*Guttera pucherani*	Crested Guineafowl	25	0	0.00 (0.00, 13.32)	14	56.00 (37.07, 73.33)	15	60.00 (40.74, 76.60)	25	100.00 (86.68, 100.00)
Numidae	*Numida meleagris*	Helmeted Guineafowl	7	0	0.00 (0.00, 35.34)	2	28.57 (8.22, 64.11)	6	85.71 (48.69, 97.43)	5	71.43 (35.89, 91.78)
		TOTAL	406	119	29.31 (25.09, 33.92)	101	24.88 (20.29, 29.30)	103	25.37 (21.38, 29.82)	251	61.82 (57.01, 66.42)

a N_samp_: Number of individual birds sampled per species.

b N_pos_: Number of haemosporidia positive birds per species.

c Prev (LCI, UCI): Prevalence estimate, upper and lower confidence interval (Brown et al., 2001).

### 2.2 Sampling

During winters, savanna habitats are characteristically water-stressed causing birds to congregate near water sources. Hence, we chose to capture granivorous birds with baited walk-in traps during the winter months of June–August of 2014. Trap design was based on the two funnel entrance traps suggested in the North American Banding Council training manual (North American Banding Council, 2012). Traps were operated between dawn (0600 h) and dusk (1800 h) and monitored with binoculars. Upon capture, we obtained between 10 and 80 μl of blood via brachial venipuncture (never exceeding 1% of the individual's body weight). Blood was collected into EDTA-coated capillary tubes to prevent coagulation, and subsequently stored in 1 ml of RNALater (Qiagen, Valencia, California), below 0 °C to preserve nucleic acids for molecular diagnostics of avian disease agents. In total, we sampled 406 birds from 3 orders, 5 families, 10 genera, and 17 species. The protocols described in this document were approved for use by the University of Florida's Institutional Animal Care and Use Committee Study No. 201408335. We did not collect biological samples from endangered or threatened species.

### 2.3 Molecular detection of haemosporidian parasites

We extracted genomic DNA from anticoagulated whole blood stored in RNALater using Qiagen DNeasy Blood and Tissue extraction kits (Qiagen, Valencia, California). We initially screened samples for haemosporidian parasites (*Plasmodium*, *Haemoproteus*, and *Leucocytozoon*) via conventional PCR using two primer sets developed for the amplification of regions on mt-*cytb* gene (Fallon et al., 2003; Beadell and Fleischer, 2005). We ran PCR reactions for both primer sets in 20 μl volumes that contained the following concentrations: 1 X reaction buffer (GoTaq Flexi Buffer, Promega, Madison, Wisconsin), 2.0 mM MgCl2 (Promega), 0.2 mM of each dNTP, 0.4 μM of each primer, and 1.0 units of Taq polymerase (Promega) and 1.5 μl of DNA template. Thermal cycling conditions were as follows: initial denaturation for 2 min at 95 °C followed by 35 cycles with 1 min denaturation at 95 °C, 1 min annealing at 58 °C (343 F/496 R) or 52 °C (213 F/372 R), extension at 72 °C for 1 min 10 s, and a final extension at 72 °C for 3 min. Suspected infections were confirmed in triplicate for each primer set. We subjected PCR-positive samples to two additional nested PCR protocols that targeted a larger fragment of the haemosporidian mt-*cytb*. We performed PCR protocols according to recommendations of Hellgren et al. (2004) and Waldenstrӧm et al. (2004). Lastly, because we sampled birds during the non-breeding season, we sexed individuals using amplification of CDH1 genes of the avian sex chromosomes (Fridolfsson and Ellegren, 1999; Griffiths et al., 1998).

We included positive and negative controls in each PCR to confirm successful amplification and non-contamination, respectively. We separated PCR products in 2% agarose gel electrophoresis stained with Gelstar (BioWhittikar Molecular Applications, Rockland, Maine) to confirm the presence of PCR products of expected length. We sequenced nested PCR products bi-directionally using dye terminator cycle sequencing on an ABI 3130 automated sequencer (Applied Biosystems, Foster City, California).

### 2.4 Identification of haemosporidia genera

To generate consensus sequences of PCR positive samples, we assembled and aligned sequence chromatograms bi-directionally using Geneious v9.1.2 (Kearse et al., 2012). We considered haemosporidian parasites with sequences differing by one or more nucleotide substitutions (≤0.2% nucleotide divergence) as evolutionary independent lineages (Ricklefs et al., 2011). We identified sequences with double peaks in the chromatograms as mixed infections, which we resolved according to methods described in Pérez-Tris and Bensch (2005). Briefly, we considered mixed infections as resolved when sequences could be matched to previously identified lineages and no double peaks were left unexplained. All unsolved mixed infections were excluded from the dataset. We identified consensus sequences to genus level (*Plasmodium*, *Haemoproteus*, *Leucocytozoon*) by comparison with published sequences available in GenBank (http://www.ncbi. nlm.nih.gov/genbank/) and the MalAvi Database (http://mbio-serv2.mbioekol.lu.se/Malavi/index.html; Bensch et al., 2009).

### 2.5 Scoring of avian life-history parameters

We categorized nest height as ground (<1 m above the ground), shrub (1–3 m above the ground), or canopy (which included subcanopy) (>3 m above the ground). We categorized nest type as open or closed. We identified the nest characteristics of brood parasitic bird species by those of the predominately parasitized host species. We categorized nest care as none (brood parasites), female-only, or shared (both sexes involved in nest care) (see Table S1). Mating system was classified as monogamous or polygynous. Lastly, we used mean tarsus length as an indicator of body size. We scored all parameters according to measurements and descriptions in The Birds of Africa series (Keith, 1992; Urban, 1986; Fry, 1992; Tarboton, 2001).

### 2.6 Statistical analyses

We used Bayesian mixed-effects models with Markov Chain Monte Carlo simulations (MCMC) to evaluate the association between life-history traits and infection with haemosporidia genera. Bayesian analyses are frequently used in epidemiological studies due to their flexibility, improved model predictions, and use of posterior probabilities that are an easily interpretable alternative to the frequentist's p-values (Dunson, 2001). We constructed regression models independently for each parasite genus (*Plasmodium*, *Haemoproteus*, *Leucocytozoon*) to predict the binary response variable (infected/uninfected) with fixed effects (nest height/nest type/nest care/mating system/body size). We included fixed effects in the models both univariately and additive multivariately (Table S3). Notably, sex was excluded from the variable set as our small sample sizes prohibited drawing meaningful conclusions between interactive effects of nest care, mating system and sex on observed prevalence differences. We controlled for host phylogenetic constraints on parasitism due to phylogenetic ancestry by including host order as a random effect. To avoid multicollinearity, we did not include traits that exhibited stronger correlations than |r| > 0.60. Following recommendations of Hadfield (2010), we used an uninformative inverse-Whishart distribution (variance, V, set to 1 and believe parameter, nu, set to 0.002). The MCMC algorithms ran for 100,000,000 iterations, with a 30% burn-in, and a sampling interval of 10,000. We assessed independency of samples in the Markov Chain via graphic diagnostics of the time series and distribution of posteriors. We restricted multivariate models to three life-history traits to ensure independency of samples and reduce over-parameterization, with the exception of models for *Plasmodium*. Due to high dependency of samples even after adjustment of iterations and sampling intervals, we limited model fitting for *Plasmodium* to univariate models. We evaluated model support using DIC (Deviance Information Criterion; Clark et al., 2016). The model with the lowest DIC statistic has the best fit to the data; we considered models differing by ≤ 2 ΔDIC units from the best-performing model as equally parsimonious. We reported estimates of posterior means with 95% lower and upper confidence intervals. We also reported posterior probabilities for the correlation between life-history traits and haemosporidia incidence, here referred to as pMCMC, the Bayesian equivalent of a p-value (Hadfield, 2010). We fit models with mixed effects implemented with the MCMCglmm function from MCMCglmm package v2.22.1 (Hadfield, 2010; Hadfield and Nakagawa, 2010); we performed model selection with function model.sel from package MuMIn v1.15.6 (Barton, 2012; R Development Core Team, 2017). We evaluated differences in infection rates between nest care and sex using Chi-square contingency table analysis in R package stats v4.0.2 (R Development Core Team, 2017).

## 3 Results

### 3.1 Prevalence of avian haemosporidia

Of 406 birds, 251 were infected with haemosporidia (61.8% prevalence). *Plasmodium* infections were most common, infecting 29.3% of all birds, while the prevalence of *Haemoproteus* and *Leucocytozoon* were 24.9% and 25.4%, respectively (Table 1). Excluding all unresolved mixed infections, 69 birds exhibited coinfections with parasites in multiple haemosporidia genera (17.0% prevalence). *Leucocytozoon* and *Haemoproteus* coinfection occurred most frequently (n_coinfected_ = 38, 55.1% of all coinfections), followed by *Leucocytozoon* and *Plasmodium* (n_coinfected_ = 29, 42.0% of all coinfections), whereas coinfection of *Plasmodium* and *Haemoproteus* was rare (n_coinfected_ = 2, 2.9% of all coinfections).

Although avian haemosporidia were prolific parasites, prevalence was comparatively low in indigobirds and whydahs (Viduidae; 7.5% prevalence). Infections with *Plasmodium* were only recovered from passerines (Passeriformes); no infections were detected in doves (Columbiformes) or guineafowl (Galliformes). Within passerines, prevalence of *Plasmodium* was highest in weavers (Ploceidae; 65.5% prevalence) and finches (Estrildidae; 56.5%); infections were least common in indigobirds and whydahs (Viduidae; 2.5%). *Haemoproteus* infections occurred in all doves (100% prevalence), were common in guineafowl (50.0%) and occurred only incidentally in passerines (17.4%), where it predominatly infected finches (30.7%) and weavers (23.2%). Guineafowl were most commonly parasitized by *Leucocytozoon* (65.6% prevalence), followed by doves (50.0%) and passerines (20.0%). In general, *Leucocytozoon* infections occurred sporadically in passerines, although some species of finches (Blue Waxbill, *Uraeginthus angolensis*) and weavers (Village Weaver, *Ploceus cucullatus*) exhibited prevalence above 35%.

We identified 76 unique haemosporidian lineages that were subsequently deposited in GenBank (Tables S3–5). Twenty-six of these lineages had previously been reported to GenBank and the MalAvi database; the remaining 50 lineages were novel. New lineages documented in this study included 44.4% of all *Plasmodium* haplotypes (n_lineage_ = 12 novel lineages), 60.0% of *Haemoproteus* haplotypes (n_lineage_ = 12), and 88.5% of *Leucocytozoon* haplotypes (n_lineage_ = 23). Two haemosporidian lineages could not be identified to genus level and were subsequently removed from further analyses.

### 3.2 Effects of life-history traits on haemosporidian parasitism

**Fig. 2:**
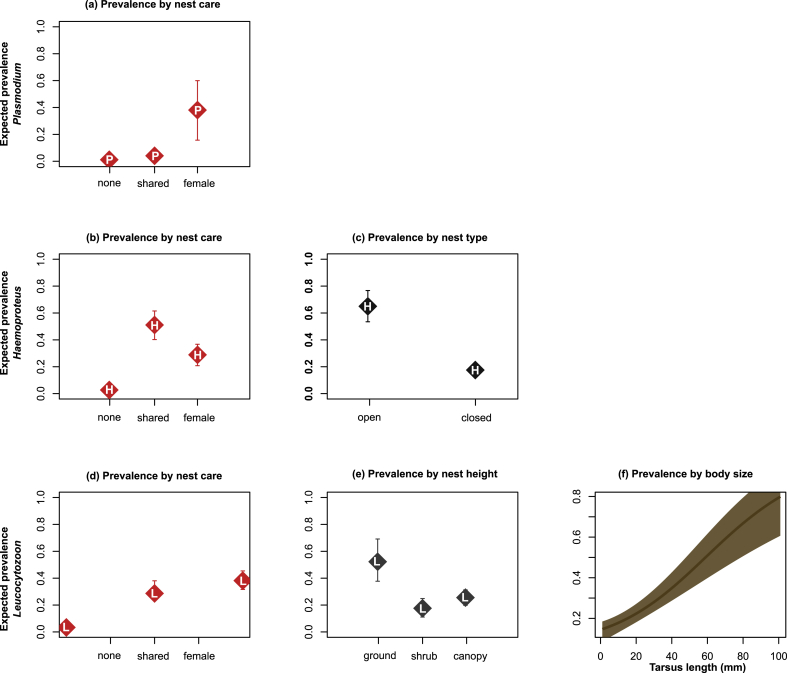
Predicted probabilities and 95% confidence intervals of haemosporidian parasitism (*Plasmodium*, *Haemoproteus*, and *Leucocytozoon*). Expected prevalence illustrated according to haemosporidia genera; *Plasmodium* represented with “P” (a), *Haemoproteus* represented with “H” (b–c), *Leucocytozoon* represented with “L” (d–f). Note that in some instances symbol size exceeded the range of confidence intervals.

Nest care was the most important predictor of infection status with haemosporidia genera (model coefficients: Table 2; model selection: Table S3). We also found that nest type was an important predictor for *Haemoproteus* infections, and that nest height and body size were important predictors for *Leucocytozoon* only (Fig. 2). Overall, the random effect due to host order contributed minimally to model fit, suggesting the effects of phylogenetic ancestry at the level of host order were low (Table 2.).

**Table 2:** Coefficients, 95% confidence intervals and p-values of best-performing models describing the association between life-history traits and haemosporidia infections.

Parameter^a^	Descrip^b^	*Plasmodium* Best-performing model's β, confidence intervals and pMCMC		*Haemoproteus* Best-performing model's beta, confidence intervals and pMCMC		*Leucocytozoon* Best-performing model's beta, confidence intervals and pMCMC	
		β (LCI, UCI)^c^	pMCMC^e^	β (LCI, UCI)	pMCMC	Β (LCI, UCI)	pMCMC
Order^f^		3.10e-1 (6.56e-4-9.96e-1)	NA	2.58e-3 (7.59e-4, 5.32e-3)	NA	2.44e-3 (7.92e-4, 4.71e-3)	NA
Nest care	none	−4.33 (−5.84, −3.12)	>**1.00e-3**	−2.46 (−3.79, −1.19)	>**1.00e-3**	−1.99 (−3.18, −0.83)	1.00e-3
	shared	−2.90 (−3.74, −2.06)	>**1.00e-3**	9.60e-1 (3.00e-1, 1.51)	>**1.00e-3**	4.82e-1 (−2.45e-1, 1.17)	2.22e-1
Nest type	open	NA	NA	1.68 (9.70e-1, 2.26)	>**1.00e-3**	NA	NA
Nest height	shrub	NA	NA	NA	NA	3.45 (2.30e-1, 7.07)	**1.43e-**2
	canopy	NA	NA	NA	NA	−1.79 (−4.21, 0.15)	5.71e-2
Body size	tarsus length	NA	NA	NA	NA	9.49e-2 (3.14e-2, 1.79e-1)	**1.00e-3**

d.LCI/UCI: Upper confidence interval/lower confidence interval.

a Parameter: Model parameters; random parameter (host order), fixed parameters (nest care, nest type, nest height, body size).

b Description: Description of life-history traits (levels).

c ß (LCI, UCI): Mean parameter estimate, upper and lower confidence interval.

e pMCMC: Posterior probability, Bayesian equivalent to p-value.

f Order: Order included in model as fixed effect.

In models for *Haemoproteus* and *Leucocytozoon*, infection probability was significantly lower in birds with no nest care compared to female or shared nest care (Fig. 2 b, d). Infections with *Haemoproteus* were highest in birds with shared nest care (n_positive_ = 43, 50.0% prevalence; Table S2); their expected rate of parasitism was 18 times higher than birds that provided no nest care (n_positive_ = 3, 2.5% prevalence). Birds with female nest care (n_positive_ = 55, 27.5% prevalence) exhibited a 10-fold increase in *Haemoproteus* infections compared to birds with no nest care. Expected rates of *Leucocytozoon* parasitism were 8-, and 12-fold greater in birds with shared (n_positive_ = 25, 29.1% prevalence) and female nest care (n_positive_ = 75, 37.5% prevalence) than birds that provide no nest care (n_positive_ = 4, 3.3% prevalence). In contrast, the probability of infection with *Plasmodium* was low in birds with no nest care (n_positive_ = 3, 2.5% prevalence) and shared nest care (n_positive_ = 6, 7.0% prevalence; Fig. 2 a) but increased nearly 10-fold in birds with female-only nest care (n_positive_ = 110, 55.0% prevalence).

Probability of parasitism by *Leucocytozoon* varied significantly by nest height. Expected rates of *Leucocytozoon* parasitism were lowest in shrub nesters (n_positive_ = 20, 17.0%) followed by canopy nesting birds (n_positive_ = 62, 25.0%), but parasitism rates nearly tripled from shrub to ground nesting birds (n_positive_ = 21, 52.5% prevalence; Fig. 2 e).

Differences in infection rates between nest care and sex were only significant for *Plasmodium*. Females with shared next care exhibited a significantly higher prevalence of *Plasmodium* than males (*X*^2^ = 7.1, p < 0.01). Males with shared nest care had a marginally higher prevalence of *Haemoproteus* than females (*X*^2^ = 3.8, p = 0.05). No sex differences were observed for *Leucocytozoon* parasites.

Body size was positively associated with the probability of parasitism by *Leucocytozoon* parasites (Fig. 2 f). Body size had no effects on infection with *Plasmodium* or *Haemoproteus*.

Nest type was an important predictor of infection with *Haemoproteus* only. We found that bird species with open nests (n_positive_ = 41, 60.1% prevalence) had a nearly 4-fold higher rate of parasitism than birds with closed nests (n_positive_ = 60, 17.5% prevalence; Fig. 2 c).

## 4 Discussion

### 4.1 Haemosporidia prevalence

We surveyed 406 savanna birds from 3 orders, 5 families, 10 genera and 17 species in northeastern Eswatini, and found a high prevalence of haemosporidia of 61.8%. Overall, our prevalence estimates are higher than in other regions of Southern Africa (Bennett et al., 1992; Okanga et al., 2015). A large-scale survey of avian haemosporidia in sub-Saharan Africa described a significantly lower prevalence in granivorous birds compared to our study (29%; Bennett et al., 1992). Infection rates from the Western Cape (South Africa) were equally low (24%; Okanga et al., 2015). However, the overall patterns of infections among host species and families were similar, with consistently higher rates of *Plasmodium* infections reported in weavers and other species of Ploceidae compared to other families (Bennett et al., 1992). In contrast to our results, the authors found that *Haemoproteus* most commonly infected birds in this region (Bennett et al., 1992). Although we found that the prevalence of *Plasmodium* was highest, differences in prevalence to *Haemoproteus* and *Leucocytozoon* were minimal, suggesting all three genera are ubiquitous parasites in the savanna avifauna (Table 1).

### 4.2 Associations between life-history traits and transmission rates of haemosporidia

Our results show that nest care was the single most important predictor of haemosporidia infections for all genera (*Plasmodium*, *Haemoproteus*, and *Leucocytozoon*). Prevalence was strikingly different between birds that provide nest care and those that do not. We found that the prevalence of haemosporidia was nearly 8-fold lower in brood parasitic passerines compared to passerines that care for their offspring. For instance, Pin-tailed Whydah (*Vidua macroura*), a common brood parasite of estrildid finches, exhibited a 5.7% prevalence while 56.5% of finches with similar life-history traits were infected with haemosporidian parasites. This pattern is consistent with the hypothesis that parental care is a costly component of reproduction (Clutton-Brock, 1991), and suggests an alternative hypothesis for the evolution of brood parasitism. The increased energetic demand of nest care likely alters host endocrinology and increases oxidative stress during this time (Fletcher et al., 2013), resulting in lower humoral and cell mediated immune responses (Derrenber et al., 1997; Moreno et al., 1999; Morales et al., 2006) which may ultimately lead to a higher disease prevalence (Nordling et al., 1998). For example, experimental manipulations of brood sizes found that males rearing enlarged brood sizes exhibited significantly higher provisioning rates and prevalence of *Plasmodium* than control groups (Richner et al., 1995).

Alternatively, nest care may increase infection risk with haemosporidian parasites due to reduced defensive behavior against host-seeking dipterans during incubation and brood care for extended periods of time (Valkiunas, 2005; Burkett-Cadena et al., 2010). Both hypotheses suggest that the abandonment of care for offspring may confer a selective advantage in environments where blood seeking parasites are abundant, and are further supported by reported positive correlations between parental effort and prevalence of diverse blood-parasites (*Plasmodium*: Richner et al., 1995; *Haemoproteus*: Allender, 1997; *Leucocytozoon*: Norris et al., 1994; *Hepatozoon*: Fargallo and Merino, 1999; *Trypanosoma*: Merino et al., 1996).

Another possible explanation for the low observed prevalence of haemosporidia in Viduidae compared to other host species examined in this study is their low population densities within savanna ecosystems in northeastern Eswatini (Monadjem, 2005). Host density has been shown to significantly affect the propagation of pathogens through host communities, where common species may be characterized by increased contact rates that ultimately result in a higher observed prevalence than rare species (Ellis et al., 2017; Jones et al., 2018). However, Fecchio et al. (2017) did not find a correlation between host density and prevalence of *Plasmodium* in avian assemblages in southern Amazonia. Similarly, Ricklefs et al. (2005) observed in avian communities of the Ozark Mountains that common as well as rare species exhibited the highest parasite prevalence. It may be speculated that in addition to effects from host density, host-specificity of avian haemosporidia also play a vital role in shaping observed patterns of prevalence. For example, rare species may lose host-specific parasite lineages (i.e. specialists) due to low contact rates that are not able to sustain transmission between generations. Preliminary evidence of a lineage analysis (Tables S3–5), suggests that loss of host-specific parasites may be a viable alternative explanation of the low prevalence of *Plasmodium* in Viduidae. *Plasmodium* lineage Swa43 (Table S3) which was incidentally recovered from brood-parasitic Long-tailed Paradise Whydah (*Vidua paradisaea*) and Dusky Indigobird (*Vidua funerea*) commonly infected Village Weavers and other species of Ploceidae. This conclusion is further supported by evidence from avian communities in South Africa where *Plasmodium* parasites were characterized by a wide host-breadth (Jones et al., 2018). However, some *Haemoproteus* and *Leucocytozoon* lineages (Swa61 and Swa09, respectively) were exclusively recovered in Viduidae, suggesting that low prevalence in these genera may not be produced by the loss of host-specific parasite lineages. Further analysis of the host community and host-specificity of haemosporidia may be necessary to disentangle these alternative scenarios.

Nest characteristics are important determinants of the prevalence of vector-borne diseases, due to differences in niche partitioning of vectors and nest structures of host species that affect the exposure rates to vectors of infectious diseases. For instance, in Malawi the incidence of haemosporidia coincides with the vertical stratification of vector species (Lutz et al., 2015). We expected that infection probability would increase with nest height, based on preferences of host-seeking dipterans for shrub and canopy level strata (Garvin and Geiner, 2003; Swanson and Adler, 2010; Černŷ et al., 2011). In contrast, in Eswatini's community of granivorous birds, the probability of haemosporidia infections particularly those of *Leucocytozoon* parasites were up to three times higher in ground-nesting birds than shrub or canopy nesters. It is worth noting here, that because many of the ground-nesting birds included in our analysis were finches, phylogeny may confound these results. We addressed this consideration by including host order as a random effect in the analysis but were unable to account for host family (Estrildidae, i.e. finches) due to small sample sizes. Although our results only partially match findings from other African bird communities, they are similar to the observations that birds nesting in lower strata are most likely to be infected with haemosporidian parasites (Fecchio et al., 2011). The responses of *Leucocytozoon* infections to nest height were unexpected as consensus suggests that blackflies (Diptera: Simuliidae) predominately host-seek at high strata (Černŷ et al., 2011). Inconsistencies between haemosporidia prevalence and nest height across studies are likely related to differences in habitat and ecology of the unique species that comprise local vector communities.

Birds with open nests were more frequently parasitized than birds that built closed nests. Infection probability with *Haemoproteus* was 4-fold higher in birds with open than closed nest types. This pattern is consistent with the Malawi avian assemblage in which closed cup nesters experienced lower rates of parasitism than birds with other nest types (Lutz et al., 2015). In contrast to vectors of other haemosporidia that predominantly rely on host-derived chemicals for host-seeking (olfactory and gustatory cues), biting midges in the genus *Culicoides* (Diptera: Ceratopogonidae) have been shown to rely on both host chemicals and visual cues to locate hosts (Bowen, 1991; Bishop et al., 2008). These results were further supported by evidence of decreased midge activity in covered areas that obstruct vision for host-seeking (Bishop, 2002). The reliance of visual cues may further explain the lower prevalence of *Haemoproteus* infection in birds that build closed nests. Contrary to an inter-regional biogeographic review of avian haemosporidia in sub-Saharan Africa, we did not find higher levels of *Haemoproteus* infections in closed-cup nesters such as weavers (Outlaw et al., 2017). According to the authors, higher prevalence in Ploceidae may be explained by the ease of hippoboscid flies and midges to move between nests of dense colonies. Although the species of Ploceidae examined in our study are colonial breeders, they are not associated with the dense colonies described elsewhere. Furthermore, Ploceidae birds were the most abundantly sampled group (Table 1) and this likely affected results of the life-history analysis due to their over-representation in the dataset.

As expected, we found that the rates of *Leucocytozoon* infections in savanna birds increased with body size; large birds such as guineafowl and doves exhibited higher prevalence than passerines. Even among passerines, larger-bodied weavers (e.g., Fan-Tailed Widowbird) were more frequently infected with these parasites compared small-bodied finches (e.g., Bronze Mannikin). Two previous studies that evaluated haemosporidia prevalence in New World avifauna reported similar responses (Ricklefs et al., 2005; González et al., 2014). The positive relationship between body size/mass and parasite prevalence may be a result of increased attraction of blood-sucking dipterans (mosquitoes: Estep et al., 2012; biting midges: Martinez-de la Puente et al., 2009; blackflies: Malmqvist et al., 2004). This pattern is consistent with the hypothesis that vectors of haemosporidia rely on host-derived chemicals such as ammonia, lactic acid and carbon dioxide to locate bloodmeals (Logan and Birkett, 2007). Large-bodied birds produce more chemical compounds which increase their attractiveness to host-seeking dipterans (Logan et al., 2010) and exposure to their disease agents (Matthews et al., 2016; Svensson-Coelho et al., 2013). In addition to increased attractiveness, large-bodied birds typically live longer and provide more surface area for vector feeding (Hamilton and Zuk, 1982), thereby increasing cumulative parasitism, and the probability of transmission of vector-borne disease.

### 4.3 Caveats and limitations

It should be considered that conclusions of this study are based on small sample sizes that limit our ability to draw definite conclusions about the prevalence patterns of avian haemosporidia observed in this study. First, we note that although we accounted for the effect of phylogenetic ancestry on the life history analysis by including host order as a random effect, we were unable to address the role of host family on prevalence. This may have confounded our conclusion of the effect of nest care. This uncertainty pertains in particular to the conclusion that brood parasitism may harbor a selective advantage. Evidence from other brood parasitic species is sparse and results are mixed. For instance, evidence based on smaller sample sizes than represented in this study suggests high infection rates with *Plasmodium* and *Haemoproteus* in brood parasitic species in the family Cuculidae (Chaisi et al., 2019). Conversely, in the eastern Baltic region *Cuculus canorus* lack infections with *Haemoproteus* and *Leucocytozoon* that characterized the host species it parasitizes (Valkiunas, 2005).

## 5 Conclusion

The analysis of life-history traits leads to a more mechanistic understanding of the link between avian life-history and transmission risk of vector-borne disease agents. In particular, the striking differences in haemosporidia prevalence between birds that provide nest care and brood parasitic birds suggest that nest care increases infection risk. Although we did not isolate the mechanisms behind this pattern, a combination of increased exposure to vectors of haemosporidia while caring for offspring on the nest, as well as increased susceptibility due to physiological changes associated with reproduction (endocrinology and oxidative stress) are likely responsible. Our analysis also highlights the importance of other host traits for the transmission of vector-borne disease agents. While the effect of traits such as nest height, nest type and body size were less consistent across haemosporidia genera, our results illustrate that differences in vector ecology and host-seeking behavior can produce variable patterns of parasitism.

## Declaration of competing interest

None.
