# Supplementary material for: Is it best on the nest? Effects of avian life-history on haemosporidian parasitism
Source: Int J Parasitol Parasites Wildl. 2020 Jul 31;13:62–71. doi: 10.1016/j.ijppaw.2020.07.014 (PMC7452475; doi:10.1016/j.ijppaw.2020.07.014)
Supplement: Multimedia component 1 [file mmc1.docx]

**Supplementary files**

**Table S1**. Life-history traits and taxonomy of granivorous birds in Eswatini. We considered the following life-history traits: Nest care (none/female/shared), body size (tarsus length in mm), mating system (monogamous/polygynous), nest type (open/closed) and nest height (ground/shrub/canopy).

| Order | Family | Scientific name | Common  Name | Nest care | Tarsus | Mating | Nest type | Nest height |
| --- | --- | --- | --- | --- | --- | --- | --- | --- |
| Passeriformes | [Estrildidae](https://en.wikipedia.org/wiki/Estrildidae) | *Lagonosticta rubricata* | African Firefinch | shared | 15.40 | monogamous | closed | ground |
| Passeriformes | [Estrildidae](https://en.wikipedia.org/wiki/Estrildidae) | *Uraeginthus angolensis* | Blue Waxbill | shared | 14.00 | monogamous | closed | shrub |
| Passeriformes | [Estrildidae](https://en.wikipedia.org/wiki/Estrildidae) | *Lonchura cucullata* | Bronze Mannikin | shared | 14.10 | monogamous | closed | canopy |
| Passeriformes | Estrildidae | *Lagonosticta senegala* | Red-billed Firefinch | shared | 11.75 | monogamous | closed | ground |
| Passeriformes | Ploceidae | *Euplectes axillaris* | Fan-tailed Widowbird | female | 22.00 | polygynous | closed | shrub |
| Passeriformes | Ploceidae | *Euplectes ardens* | Red-collared Widowbird | female | 21.00 | polygynous | closed | canopy |
| Passeriformes | Ploceidae | *Euplectes orix* | Red Bishop | female | 21.50 | polygynous | open | shrub |
| Passeriformes | Ploceidae | *Ploceus cucullatus* | Village Weaver | female | 22.40 | polygynous | closed | canopy |
| Passeriformes | Viduidae | *Vidua paradisaea* | Long-tailed Paradise Whydah | none | 17.00 | polygynous | closed | shrub |
| Passeriformes | Viduidae | *Vidua macroura* | Pin-tailed Whydah | none | 16.5 | polygynous | closed | shrub |
| Passeriformes | Viduidae | *Vidua funerea* | Dusky Indigobird | none | 13.00 | polygynous | closed | canopy |
| Passeriformes | Viduidae | *Vidua chalybeata* | Village Indigobird | none | 15.71 | polygynous | closed | shrub |
| Columbiformes | Columbidae | *Streptopelia capicola* | Cape Turtle Dove | shared | 21.40 | monogamous | open | canopy |
| Columbiformes | Columbidae | *Turtur chalcospilos* | Emerald-spotted Wood Dove | shared | 18.00 | monogamous | open | canopy |
| Columbiformes | Columbidae | *Streptopelia semitorquata* | Red-eyed Dove | shared | 24.60 | monogamous | open | canopy |
| Galliformes | Numidae | *Guttera pucherani* | Crested Guineafowl | female | 89.50 | monogamous | open | ground |
| Galliformes | Numidae | *Numida meleagris* | Helmeted Guineafowl | female | 82.00 | monogamous | open | ground |

**Table S2**. Prevalence of haemosporidia by life-history traits of granivorous birds in Eswatini. Table indicates Plasmodium, Haemoproteus and Leucocytozoon prevalence estimates (Wilson score interval; Brown et al. 2001).

| Life-history trait | N_samp_^1^ | *Plasmodium* | | *Haemoproteus* | | *Leucocytozoon* | |
| --- | --- | --- | --- | --- | --- | --- | --- |
|  |  | N_pos_^2^ | % Prev (LCI, UCI)3 | N_pos_ | % Prev (LCI, UCI) | N_pos_ | % Prev (LCI, UCI) |
| **Mating system** |  |  | |  | |  | |
| monogamous | 118 | 6 | 5.09 (2.35, 10.65) | 59 | 50.00 (41.12, 58.88) | 45 | 38.17 (29.88, 47.14) |
| polygynous | 288 | 113 | 39. 24 (33.77, 44.98) | 42 | 14.58 (10.97, 19.13) | 58 | 20.14 (15.91, 25.15) |
| **Nest care** |  |  |  |  |  |  |  |
| none | 120 | 3 | 2.50 (0.85, 7.09) | 3 | 2.50 (0.85, 7.09) | 4 | 3.33 (1.30, 8.26) |
| shared | 86 | 6 | 6.98 (3.24, 14.40) | 43 | 50.00 (39.67, 60.40) | 25 | 29.07 (20.53, 39.40) |
| female | 200 | 110 | 55.00 (48.08, 61.74) | 55 | 27.50 (21.78, 34.07) | 75 | 37.50 (31.09, 44.39) |
| **Nest type** |  |  |  |  |  |  |  |
| open | 63 | 5 | 7.94 (3.44, 17.27) | 41 | 60.08 (52.75, 75.67) | 34 | 53.97 (41.79, 65.69) |
| closed | 343 | 114 | 33.24 (28.46, 38.38) | 60 | 17.49 (13.84, 21.87) | 69 | 20.12 (16.22, 24,68) |
| **Nest height** |  |  |  |  |  |  |  |
| ground | 40 | 2 | 5.00 (1.38, 16.50) | 16 | 40.00 (26.35, 55.40) | 21 | 52.50 (37.50, 67.07) |
| shrub | 118 | 36 | 30.51 (22.93, 39.32) | 21 | 17.78 (11.95, 25.68) | 20 | 16.95 (11.25, 24.73) |
| canopy | 248 | 81 | 32.66 (27.13, 38,72) | 64 | 25.81 (20.76, 31.59) | 62 | 25.00 (20.02, 30.74) |

^1^N_samp_: Number of individuals birds sampled per species

^2^N_pos_: Number of haemosporidia positive birds per species

^3^Prev (LCI, UCI): Prevalence estimate, upper and lower confidence interval (Brown et al. 2001)

**Table S3**. Set of four top-performing Bayesian mixed-effects models for the association between *Plasmodium* incidence and life-history traits of savanna birds in Eswatini.

| Haemosporidian  Genus | M^a^ | Nest care^b^ | Body size^c^ | Mating^d^ | Nest type^e^ | Nest height^f^ | DF^g^ | DIC^h^ | ∆DIC^i^ | DIC weight |
| --- | --- | --- | --- | --- | --- | --- | --- | --- | --- | --- |
| *Plasmodium* | 1 | X |  |  |  |  | 5 | 322.09 | 0.00 | 1.00 |
|  | 2 |  |  | X |  |  | 4 | 437.09 | 115.00 | 0.00 |
|  | 3 |  |  |  | X |  | 4 | 475.01 | 152.93 | 0.00 |
|  | 4 |  |  |  |  | X | 5 | 479.73 | 157.65 | 0.00 |
| *Haemoproteus* | 1 | X |  |  | X |  | 6 | 360.28 | 0.00 | 0.59 |
|  | 2 | X | X |  | X |  | 7 | 361.00 | 0.72 | 0.41 |
|  | 3 | X | X |  |  |  | 6 | 376.02 | 15.74 | 0.00 |
|  | 4 | X |  |  |  |  | 5 | 384.62 | 24.34 | 0.00 |
| *Leucocytozoon* | 1 | X | X |  |  | X | 8 | 392.62 | 0.00 | 0.52 |
|  | 2 | X |  |  | X |  | 6 | 395.01 | 2.28 | 0.16 |
|  | 3 | X | X |  | X |  | 7 | 395.51 | 2.87 | 0.12 |
|  | 4 | X | X |  |  |  | 6 | 395.56 | 2.93 | 0.12 |

Set of candidate models and the relative support of the data for these models (∆DIC values) from DIC-based model comparison. We used these comparisons to identify characteristics associated with *Plasmodium* incidence in savanna birds.

^a^M = model

^b^Nest care = classified as: none, female, shared

^c^Body size = body size inferred from tarsus measurements (mm)

^d^Mating = mating system, classified as: monogamous or polygynous

^e^Nest type = classified as open, closed

^f^Nest height = classified as ground, shrub, canopy/subcanopy level

^g^DF = degrees of freedom

^h^DIC = Deviance Information Criterion

^i^∆DIC = change in Deviance Information Criterion

**Table S3**. Distribution of *Plasmodium* haplotypes among host species. Detection and non-detection of *Plasmodium* haplotypes by species indicated with 1 and 0, respectively.

| Hap^1^  (Genbank Accession) | Estrildidae | | | | Ploceidae | | | | Viduidae | | | | Columbidae | | | Numidae | |
| --- | --- | --- | --- | --- | --- | --- | --- | --- | --- | --- | --- | --- | --- | --- | --- | --- | --- |
|  | AF  FI | BL  WA | BR  MA | RB  FF | FT  WB | RC  WB | RE  BI | VI  WE | LT  PW | PT  WH | DU  IN | VI IN | CT  DO | ES  WD | RE  DO | CR  GU | HE  GU |
| Swa29 (MT761642) | 0 | 0 | 0 | 0 | 1 | 1 | 1 | 0 | 0 | 0 | 0 | 0 | 0 | 0 | 0 | 0 | 0 |
| Swa30 (MT761643) | 0 | 0 | 0 | 0 | 0 | 0 | 0 | 1 | 0 | 0 | 0 | 0 | 0 | 0 | 0 | 0 | 0 |
| Swa31 (MT761644) | 0 | 0 | 0 | 0 | 1 | 0 | 1 | 1 | 0 | 0 | 0 | 0 | 0 | 0 | 0 | 0 | 0 |
| Swa32 (MT761645) | 0 | 0 | 0 | 0 | 0 | 0 | 0 | 1 | 0 | 0 | 0 | 0 | 0 | 0 | 0 | 0 | 0 |
| Swa33 (MT761646) | 0 | 0 | 0 | 0 | 0 | 0 | 0 | 1 | 0 | 0 | 0 | 0 | 0 | 0 | 0 | 0 | 0 |
| Swa34 (MT761647) | 0 | 0 | 0 | 0 | 1 | 0 | 0 | 1 | 0 | 0 | 0 | 0 | 0 | 0 | 0 | 0 | 0 |
| Swa35 (MT761648) | 0 | 0 | 1 | 0 | 0 | 0 | 0 | 1 | 0 | 0 | 0 | 0 | 0 | 0 | 0 | 0 | 0 |
| Swa36 (MT761649) | 0 | 0 | 0 | 0 | 1 | 0 | 0 | 0 | 0 | 0 | 0 | 0 | 0 | 0 | 0 | 0 | 0 |
| Swa37 (MT761650 | 0 | 0 | 0 | 0 | 1 | 0 | 0 | 0 | 0 | 0 | 0 | 0 | 0 | 0 | 0 | 0 | 0 |
| Swa38 (MT761651) | 0 | 0 | 0 | 0 | 1 | 0 | 0 | 1 | 0 | 0 | 0 | 0 | 0 | 0 | 0 | 0 | 0 |
| Swa39 (MT761652) | 0 | 0 | 0 | 0 | 0 | 0 | 0 | 1 | 0 | 0 | 0 | 0 | 0 | 0 | 0 | 0 | 0 |
| Swa43 (MT761655) | 0 | 0 | 0 | 0 | 0 | 1 | 1 | 1 | 1 | 0 | 1 | 0 | 0 | 0 | 0 | 0 | 0 |
| Swa44 (MT761656) | 0 | 0 | 0 | 0 | 0 | 0 | 0 | 1 | 0 | 0 | 0 | 0 | 0 | 0 | 0 | 0 | 0 |
| Swa45 (MT761657) | 0 | 1 | 0 | 1 | 1 | 0 | 0 | 1 | 0 | 0 | 0 | 0 | 0 | 0 | 0 | 0 | 0 |
| Swa46 (MT761658) | 0 | 0 | 0 | 0 | 0 | 0 | 0 | 1 | 0 | 0 | 0 | 0 | 0 | 0 | 0 | 0 | 0 |
| Swa47 (MT761659) | 0 | 0 | 0 | 0 | 0 | 0 | 0 | 1 | 0 | 0 | 0 | 0 | 0 | 0 | 0 | 0 | 0 |
| Swa48 (MT761660) | 0 | 0 | 0 | 0 | 0 | 0 | 0 | 1 | 0 | 0 | 0 | 0 | 0 | 0 | 0 | 0 | 0 |
| Swa49 (MT761661) | 0 | 0 | 0 | 0 | 0 | 0 | 0 | 1 | 0 | 0 | 0 | 0 | 0 | 0 | 0 | 0 | 0 |
| Swa63 (MT761674) | 0 | 1 | 0 | 0 | 1 | 0 | 0 | 1 | 0 | 0 | 0 | 0 | 0 | 0 | 0 | 0 | 0 |
| Swa64 (MT761675) | 0 | 0 | 0 | 0 | 0 | 0 | 0 | 1 | 0 | 0 | 0 | 0 | 0 | 0 | 0 | 0 | 0 |
| Swa66 (MT761677) | 0 | 0 | 0 | 0 | 1 | 0 | 0 | 0 | 0 | 0 | 0 | 0 | 0 | 0 | 0 | 0 | 0 |
| Swa67 (MT761678) | 0 | 0 | 0 | 1 | 0 | 0 | 0 | 1 | 0 | 0 | 0 | 0 | 0 | 0 | 0 | 0 | 0 |
| Swa68 (MT761679) | 0 | 0 | 0 | 0 | 0 | 0 | 0 | 1 | 0 | 0 | 0 | 0 | 0 | 0 | 0 | 0 | 0 |
| Swa69 (MT761680) | 0 | 0 | 0 | 0 | 0 | 0 | 0 | 1 | 0 | 0 | 0 | 0 | 0 | 0 | 0 | 0 | 0 |
| Swa70 (MT761681) | 0 | 0 | 0 | 0 | 1 | 0 | 0 | 0 | 0 | 0 | 0 | 0 | 0 | 0 | 0 | 0 | 0 |
| Swa72 (MT761683) | 0 | 0 | 0 | 0 | 1 | 0 | 0 | 0 | 0 | 0 | 0 | 0 | 0 | 0 | 0 | 0 | 0 |
| Swa73 (MT761684) | 0 | 0 | 0 | 0 | 1 | 0 | 0 | 0 | 0 | 0 | 0 | 0 | 0 | 0 | 0 | 0 | 0 |
| Swa74 (MT761685) | 0 | 0 | 0 | 0 | 0 | 0 | 0 | 1 | 0 | 0 | 0 | 0 | 0 | 0 | 0 | 0 | 0 |

^1^Hap = Haplotype
Species Abbreviations: AFFI (African Firefinch), BLWA (Blue Waxbill), BRMA (Bronze Mannikin), RBFF (Red-billed Firefinch), FTWB (Fan-tailed Widowbird), RCWB (Red-collared Widowbird), REBI (Red Bishop), VIWE (Village Weaver), LTPW (Long-tailed Paradise Whydah), PTWH (Pin-tailed Whydah), DUIN (Dusky Indigobird), VIIN (Village Indigobird), CTDO (Cape Turtle Dove), ESWD (Emerald-spotted Wood Dove), REDO (Red-eyed Dove), CRGU (Crested Guineafowl), and HEGU (Helmeted Guineafowl)

**Table S4**. Distribution of *Haemoproteus* (including *Parahaemoproteus*) haplotypes among host species. Detection and non-detection of *Haemoproteus* haplotypes by species indicated with 1 and 0, respectively.

| Hap^1^  (Genbank Accession) | Estrildidae | | | | Ploceidae | | | | Viduidae | | | | Columbidae | | | Numidae | |
| --- | --- | --- | --- | --- | --- | --- | --- | --- | --- | --- | --- | --- | --- | --- | --- | --- | --- |
|  | AF  FI | BL  WA | BR  MA | RB  FF | FT  WB | RC  WB | RE  BI | VI  WE | LT  PW | PT  WH | DU  IN | VI  IN | CT  DO | ES  WD | RE  DO | CR  GU | HE  GU |
| Swa27 (MT761640) | 0 | 0 | 0 | 0 | 0 | 0 | 0 | 1 | 0 | 0 | 0 | 0 | 0 | 0 | 0 | 0 | 0 |
| Swa28 (MT761641) | 0 | 0 | 0 | 0 | 0 | 0 | 0 | 1 | 0 | 0 | 0 | 0 | 0 | 0 | 0 | 0 | 0 |
| Swa40 (MT761641) | 0 | 0 | 0 | 0 | 0 | 0 | 0 | 1 | 0 | 0 | 0 | 0 | 0 | 0 | 0 | 0 | 0 |
| Swa41 (MT761654) | 0 | 0 | 0 | 0 | 0 | 0 | 0 | 1 | 0 | 0 | 0 | 0 | 0 | 0 | 0 | 0 | 0 |
| Swa50 (MT761662) | 0 | 1 | 0 | 0 | 0 | 0 | 0 | 0 | 0 | 0 | 0 | 0 | 0 | 0 | 1 | 0 | 0 |
| Swa51 (MT761663) | 0 | 0 | 0 | 0 | 0 | 0 | 0 | 0 | 0 | 0 | 0 | 0 | 0 | 0 | 1 | 0 | 0 |
| Swa52 (MT761664) | 0 | 0 | 0 | 0 | 0 | 0 | 0 | 0 | 0 | 0 | 0 | 0 | 0 | 1 | 0 | 0 | 0 |
| Swa53 (MT761665) | 0 | 0 | 0 | 0 | 0 | 0 | 0 | 0 | 0 | 0 | 0 | 0 | 0 | 0 | 0 | 1 | 0 |
| Swa54 (MT761666) | 0 | 0 | 0 | 0 | 0 | 0 | 0 | 0 | 0 | 0 | 0 | 0 | 0 | 0 | 0 | 1 | 0 |
| Swa55 (MT761667) | 0 | 0 | 0 | 0 | 0 | 0 | 0 | 0 | 0 | 0 | 0 | 0 | 0 | 0 | 0 | 1 | 0 |
| Swa57 (MT761668) | 0 | 0 | 0 | 0 | 0 | 0 | 0 | 0 | 0 | 0 | 0 | 0 | 0 | 1 | 0 | 0 | 0 |
| Swa58 (MT761669) | 0 | 0 | 0 | 0 | 0 | 0 | 0 | 0 | 0 | 0 | 0 | 0 | 0 | 1 | 0 | 0 | 0 |
| Swa59 (MT761670) | 0 | 0 | 0 | 0 | 0 | 0 | 0 | 0 | 0 | 0 | 0 | 0 | 1 | 0 | 1 | 0 | 0 |
| Swa60 (MT761671) | 0 | 1 | 1 | 0 | 1 | 0 | 0 | 0 | 0 | 0 | 0 | 0 | 0 | 0 | 0 | 0 | 0 |
| Swa61 (MT761672) | 0 | 0 | 0 | 0 | 0 | 0 | 0 | 0 | 0 | 1 | 1 | 0 | 0 | 0 | 0 | 0 | 0 |
| Swa62 (MT761673) | 0 | 0 | 0 | 0 | 0 | 0 | 1 | 0 | 0 | 0 | 0 | 0 | 0 | 0 | 0 | 0 | 0 |
| Swa65 (MT761674) | 0 | 0 | 0 | 0 | 0 | 0 | 0 | 1 | 0 | 0 | 0 | 0 | 0 | 0 | 0 | 0 | 0 |
| Swa71 (MT761682) | 0 | 0 | 0 | 0 | 0 | 0 | 0 | 1 | 0 | 0 | 0 | 0 | 0 | 0 | 0 | 0 | 0 |
| Swa75 (MT761686) | 0 | 0 | 0 | 0 | 0 | 0 | 0 | 0 | 0 | 0 | 0 | 0 | 0 | 0 | 0 | 0 | 1 |
| Swa77 (MT761687) | 0 | 1 | 0 | 0 | 0 | 0 | 0 | 0 | 0 | 0 | 0 | 0 | 0 | 0 | 0 | 0 | 0 |

^1^Hap = Haplotype
Species Abbreviations: AFFI (African Firefinch), BLWA (Blue Waxbill), BRMA (Bronze Mannikin), RBFF (Red-billed Firefinch), FTWB (Fan-tailed Widowbird), RCWB (Red-collared Widowbird), REBI (Red Bishop), VIWE (Village Weaver), LTPW (Long-tailed Paradise Whydah), PTWH (Pin-tailed Whydah), DUIN (Dusky Indigobird), VIIN (Village Indigobird), CTDO (Cape Turtle Dove), ESWD (Emerald-spotted Wood Dove), REDO (Red-eyed Dove), CRGU (Crested Guineafowl), and HEGU (Helmeted Guineafowl)

**Table S4**. Distribution of *Leucocytozoon* haplotypes among host species. Detection and non-detection of *Leucocytozoon* haplotypes by species indicated with 1 and 0, respectively.

| Hap^1^  (Genbank Accession) | Estrildidae | | | | Ploceidae | | | | Viduidae | | | | Columbidae | | | Numidae | |
| --- | --- | --- | --- | --- | --- | --- | --- | --- | --- | --- | --- | --- | --- | --- | --- | --- | --- |
|  | AF  FI | BL  WA | BR  MA | RB  FF | FT  WB | RC  WB | RE  BI | VI  WE | LT  PW | PT  WH | DU  IN | VI  IN | CT  DO | ES  WD | RE  DO | CR  GU | HE  GU |
| Swa01 (MT761614) | 0 | 0 | 0 | 0 | 0 | 0 | 0 | 0 | 0 | 0 | 0 | 0 | 0 | 0 | 0 | 1 | 0 |
| Swa02 (MT761615) | 0 | 0 | 0 | 0 | 0 | 0 | 0 | 0 | 0 | 0 | 0 | 0 | 0 | 0 | 0 | 1 | 0 |
| Swa03 (MT761616) | 0 | 0 | 0 | 0 | 0 | 0 | 0 | 0 | 0 | 0 | 0 | 0 | 0 | 1 | 1 | 0 | 0 |
| Swa04 (MT761617) | 0 | 0 | 0 | 0 | 0 | 0 | 0 | 0 | 0 | 0 | 0 | 0 | 0 | 0 | 1 | 0 | 0 |
| Swa05 (MT761618) | 0 | 0 | 0 | 0 | 0 | 0 | 0 | 0 | 0 | 0 | 0 | 0 | 0 | 0 | 1 | 0 | 0 |
| Swa06 (MT761619) | 0 | 1 | 0 | 0 | 1 | 0 | 0 | 1 | 0 | 0 | 0 | 0 | 0 | 0 | 0 | 0 | 1 |
| Swa07 (MT761620) | 0 | 1 | 0 | 0 | 0 | 0 | 0 | 0 | 0 | 0 | 0 | 0 | 0 | 0 | 0 | 0 | 0 |
| Swa08 (MT761621) | 0 | 1 | 0 | 0 | 1 | 0 | 0 | 1 | 0 | 0 | 0 | 0 | 0 | 0 | 0 | 0 | 0 |
| Swa09 (MT761622) | 0 | 0 | 0 | 0 | 0 | 0 | 0 | 0 | 0 | 0 | 1 | 0 | 0 | 0 | 0 | 0 | 0 |
| Swa10 (MT761623) | 0 | 0 | 0 | 0 | 0 | 0 | 0 | 1 | 0 | 0 | 1 | 0 | 0 | 0 | 0 | 0 | 0 |
| Swa11 (MT761624) | 0 | 0 | 0 | 0 | 0 | 0 | 0 | 1 | 0 | 0 | 1 | 0 | 0 | 0 | 0 | 0 | 0 |
| Swa12 (MT761625) | 0 | 0 | 0 | 0 | 0 | 0 | 0 | 1 | 0 | 0 | 0 | 0 | 0 | 0 | 0 | 0 | 0 |
| Swa13 (MT761626) | 0 | 1 | 0 | 0 | 0 | 0 | 0 | 1 | 0 | 0 | 0 | 0 | 0 | 0 | 0 | 0 | 0 |
| Swa14 (MT761627) | 0 | 0 | 0 | 0 | 0 | 0 | 0 | 1 | 0 | 0 | 0 | 0 | 0 | 0 | 0 | 0 | 0 |
| Swa15 (MT761628) | 0 | 0 | 0 | 0 | 0 | 0 | 0 | 1 | 0 | 0 | 0 | 0 | 0 | 0 | 0 | 0 | 0 |
| Swa16 (MT761629) | 0 | 0 | 0 | 0 | 0 | 0 | 0 | 1 | 0 | 0 | 0 | 0 | 0 | 0 | 0 | 0 | 0 |
| Swa17 (MT761630) | 0 | 0 | 0 | 0 | 0 | 0 | 0 | 0 | 0 | 0 | 0 | 0 | 0 | 0 | 0 | 0 | 1 |
| Swa18 (MT761631) | 0 | 0 | 0 | 0 | 0 | 0 | 0 | 0 | 0 | 0 | 0 | 0 | 0 | 0 | 0 | 0 | 1 |
| Swa19 (MT761632) | 0 | 1 | 0 | 0 | 0 | 0 | 0 | 0 | 0 | 0 | 0 | 0 | 0 | 0 | 0 | 0 | 0 |
| Swa20 (MT761633) | 0 | 1 | 0 | 0 | 0 | 0 | 0 | 0 | 0 | 0 | 0 | 0 | 0 | 0 | 0 | 0 | 0 |
| Swa21 (MT761634) | 0 | 1 | 0 | 0 | 0 | 0 | 1 | 0 | 0 | 0 | 0 | 0 | 0 | 0 | 0 | 0 | 0 |
| Swa22 (MT761635) | 0 | 1 | 0 | 0 | 0 | 0 | 0 | 0 | 0 | 0 | 0 | 0 | 0 | 0 | 0 | 0 | 0 |
| Swa23 (MT761636) | 0 | 0 | 0 | 0 | 0 | 0 | 1 | 0 | 0 | 0 | 0 | 0 | 0 | 0 | 0 | 0 | 0 |
| Swa24 (MT761637) | 0 | 0 | 0 | 0 | 0 | 0 | 0 | 1 | 0 | 0 | 0 | 0 | 0 | 0 | 0 | 0 | 0 |
| Swa25 (MT761638) | 0 | 0 | 0 | 0 | 0 | 0 | 0 | 1 | 0 | 0 | 0 | 0 | 0 | 0 | 0 | 0 | 0 |
| Swa26 (MT761639) | 0 | 0 | 0 | 0 | 0 | 0 | 0 | 1 | 0 | 0 | 0 | 0 | 0 | 0 | 0 | 0 | 0 |

^1^Hap = Haplotype
Species Abbreviations: AFFI (African Firefinch), BLWA (Blue Waxbill), BRMA (Bronze Mannikin), RBFF (Red-billed Firefinch), FTWB (Fan-tailed Widowbird), RCWB (Red-collared Widowbird), REBI (Red Bishop), VIWE (Village Weaver), LTPW (Long-tailed Paradise Whydah), PTWH (Pin-tailed Whydah), DUIN (Dusky Indigobird), VIIN (Village Indigobird), CTDO (Cape Turtle Dove), ESWD (Emerald-spotted Wood Dove), REDO (Red-eyed Dove), CRGU (Crested Guineafowl), and HEGU (Helmeted Guineafowl)
